# mHealth interventions to reduce maternal and child mortality in Sub-Saharan Africa and Southern Asia: A systematic literature review

**DOI:** 10.3389/fgwh.2022.942146

**Published:** 2022-08-25

**Authors:** Elvis Bossman, Monika A. Johansen, Paolo Zanaboni

**Affiliations:** ^1^Department of Clinical Medicine, UiT The Arctic University of Norway, Tromsø, Norway; ^2^Norwegian Centre for E-Health Research, University Hospital of North Norway, Tromsø, Norway

**Keywords:** mHealth, maternal health, child health, antenatal care, post-natal care, Sustainable Development Goals, low and middle-income countries

## Abstract

**Background:**

Reducing maternal mortality, neonatal mortality and under 5-year mortality are important targets addressed by the United Nations' Sustainable Development Goals. Despite studies reported an improvement in maternal and child health indicators, the progress achieved is not uniform across regions. Due to the increasing availability of mobile phones in low and middle-income countries, mHealth could impact considerably on reducing maternal and child mortality and maximizing women's access to quality care, from the antenatal stage to the post-natal period.

**Methods:**

A systematic literature review of mHealth interventions aimed at reducing maternal and child mortality in Sub-Saharan Africa and Southern Asia. Primary outcomes were maternal mortality, neonatal mortality, and under-five mortality. Secondary outcomes were skilled birth attendance, antenatal care (ANC) and post-natal care (PNC) attendance, and vaccination/immunization coverage. We searched for articles published from January 2010 to December 2020 in Embase, Medline and Web of Science. Quantitative comparative studies were included. The protocol was developed according to the PRISMA Checklist and published in PROSPERO [CRD42019109434]. The Quality Assessment Tool for Quantitative Studies was used to assess the quality of the eligible studies.

**Results:**

23 studies were included in the review, 16 undertaken in Sub-Saharan Africa and 7 in Southern Asia. Most studies used SMS or voice message reminders for education purposes. Only two studies reported outcomes on neonatal mortality, with positive results. None of the studies reported results on maternal mortality or under-five mortality. Outcomes on skilled birth attendance, ANC attendance, PNC attendance, and vaccination coverage were reported in six, six, five, and eleven studies, respectively. Most of these studies showed a positive impact of mHealth interventions on the secondary outcomes.

**Conclusion:**

Simple mHealth educational interventions based on SMS and voice message reminders are effective at supporting behavior change of pregnant women and training of health workers, thus improving ANC and PNC attendance, vaccination coverage and skilled birth attendance. Higher quality studies addressing the role of mHealth in reducing maternal and child mortality in resource-limited settings are needed, especially in Southern Asia.

**Systematic review registration:**

https://www.crd.york.ac.uk/prospero/display_record.php?ID=CRD42019109434, identifier CRD42019109434.

## Introduction

The Millennium Development Goals (MDGs) were established in 2000 after the Millennium Summit of the United Nations (UN) (1, 2). Child and maternal mortality, addressed by MDG 4 and MDG 5, respectively, experienced a substantial decline from 2000 to 2015 (3–6). The global under 5-year mortality was reduced from 11.9 million deaths in 1990 to 7.7 million deaths in 2010 (7), with an accelerated decline reported during the second decade. Despite the target of decreasing maternal mortality by 75% was not achieved, all MDG regional groupings showed a significant improvement (4, 5). The end of the MDG era in 2015 marked the transition into the Sustainable Development Goals (SDGs), which encompassed global targets of further reducing maternal mortality, neonatal mortality and under 5-year mortality (8). The SDGs, implemented with another 15-year plan due by 2030, represent a comprehensive blueprint for sustainable development toward health and wellbeing. Over 50 indicators are defined by the SDGs to measure health outcomes and health service delivery (8). Reproductive, maternal, newborn and child health are among the main thematic areas.

Several studies reported an improvement in maternal and child health indicators (4, 7). Nevertheless, a UN report on the MDGs evaluations indicated that, regardless of the progress achieved, the reduction in maternal and child mortality across most regions was not uniform (2). This culminated in the inability to achieve the MDGs. According to Solow's growth model (9), a greater progress among low-income countries compared with high-income countries leads to convergence across the world countries. On the contrary, a greater progress among high-income countries widens the gaps, leading to overall divergence among countries (2, 9). There is therefore a call for a strong program of action and interventions to bring equity and eliminate the divergent progress on maternal and child health between high-income and low-income countries. Interventions aimed at eradicating preventable maternal and child mortality are required in resource-limited settings, such as Sub-Saharan Africa and Southern Asia, to ensure convergent progress worldwide.

Since most people in low and middle-income countries have a mobile phone (10), interventions based on mobile technologies represent a very promising tool to increase efficiency in health care and enhance service utilization (11–14). Mobile health (mHealth) is defined as medical and public health practice supported by mobile devices, such as mobile phones, patient monitoring devices, personal digital assistants, and other wireless devices (15). Such solutions enable the provision of healthcare anytime and anywhere, overcoming organizational and geographical barriers. mHealth has the potential to address the health challenges and needs of the Global South, particularly in Sub-Saharan Africa and Southern Asia (16–18). The introduction of mHealth interventions could impact considerably on reducing maternal mortality (11) and maximizing women's access to quality care (19), from the antenatal stage (20, 21) to the post-natal period (11, 22–24). mHealth might support the global effort in addressing the SDG 3 and achieving the SDG Target 3.1 and 3.2 on maternal and neonatal/child health, respectively (25, 26).

This study aimed to evaluate the impact of mHealth interventions on maternal and child health in resource-limited settings. The primary outcomes directly related to SDG Target 3.1 and 3.2 were: (1) maternal mortality (SDG Indicator 3.1.1), (2) neonatal mortality (SDG Indicator 3.2.2), (3) under-five mortality (SDG Indicator 3.2.1). Secondary outcomes selected by the authors due to their impact on maternal and child mortality included: skilled birth attendance (SDG Indicator 3.1.2), antenatal care (ANC) and post-natal care (PNC) attendance, vaccination/immunization coverage. The identification of effective mHealth interventions could contribute to an accelerated progress toward achieving the SDG 3 by 2030.

## Methods

### Study design

We performed a systematic literature review (27) on mHealth interventions aimed at reducing preventable maternal, neonatal and under-five mortality in Sub-Saharan Africa and Southern Asia. The review was conducted in accordance with the Preferred Reporting Items for Systematic review and Meta-Analysis Protocols (PRISMA-P) (28, 29) to ensure a transparent and complete reporting of the study. The study protocol was developed a priori and published in the International Prospective Register of Systematic Reviews (PROSPERO) [registration number CRD42019109434] (30).

### Eligibility criteria

The scope of the review and eligibility criteria were formulated using the PICOS framework (participants, interventions, comparisons, outcomes, study type) (31) (Table 1).

**Table 1:** Eligibility criteria.

**Study type:** Quantitative comparative studies, including randomized controlled trials, non-randomized control trials, case-controlled trials, and pre-post study designs.
**Population:** Studies on pregnant women or women in their post-natal period and/or children under 5 years including newborns. Studies on interventions implemented in health care facilities or for health care workers with outcomes on maternal and child health.
**Interventions:** Studies focused primarily on mHealth interventions for maternal health and child health in Sub-Saharan Africa or Southern Asia. Studies where mHealth was a co-intervention were excluded.
**Outcomes:** Studies with outcomes addressing the SDG Target 3.1 and 3.2: maternal mortality, neonatal mortality and under 5-year mortality. Studies with secondary outcomes which impact on maternal mortality and child mortality: skilled birth attendance, antenatal care and post-natal care attendance, vaccination/immunization coverage.
**Setting:** Studies conducted or implemented in Sub-Saharan Africa or Southern Asia as defined by the World Bank's country classification.
**Language:** Studies with full-text available in English.
**Publication date**: Studies published between January 2010 and December 2020 (data extraction).
**Risk assessment:** Studies with a methodological rating of STRONG or MODERATE according to the Quality Assessment Tool for Quantitative Studies.

### Data sources and search strategy

Relevant articles were searched in Medline, Embase and Web of Science. The search strategy was based on the PICOS framework and developed using selected keywords as well as thesaurus terms of each database. The search strategy (Supplementary Table 1) included terms relating to or describing mHealth interventions according to the eligibility criteria. The search strategy was tested before starting the formal screening. The results of each database search were stored into a single reference database (Endnote). Duplicate references were removed. The electronic search was performed by one review team member (EB).

### Study selection and data extraction

Titles and abstracts were screened, and studies which did not meet the inclusion criteria were excluded. The full texts of the selected studies were then retrieved and independently assessed for eligibility by two review team members (EB, PZ). Any disagreement over the eligibility of particular studies was resolved through discussion and the involvement of another reviewer (MJ). A standardized data extraction form was developed, piloted and used to extract information from the full texts for evidence synthesis (Supplementary Table 2). For each article, results were extracted for the primary and secondary outcomes (Table 2).

**Table 2:** Definition of the study outcomes.

**Primary outcomes**
*Maternal mortality (deaths)*. The death of a woman while pregnant or within 42 days of termination of pregnancy, from any cause related to or aggravated by the pregnancy or its management (from direct or indirect obstetric death), but not from accidental or incidental causes in the gestation period and childbirth or within 42 days after termination of pregnancy regardless of the length and site of the pregnancy.
*Neonatal mortality (deaths)*. The death of a newborn during the first 28 days of life. In 2017, ~2.5 million deaths occurred in the first month of life. On average, 7,000 deaths occurred every day, the majority of which happening in the first week after birth. Around 36% of deaths occurred the same day of birth, and about 75% of all newborn deaths occurred in the first week of life. The global neonatal mortality rate fell from 37 deaths per 1,000 live births in 1990 to 18 in 2017.
*Under-five mortality (deaths)*. The death of a child during the first 5 years of life. Worldwide, most child and young adolescent deaths happen at the youngest ages. In 2017, 85% of the 6.3 million deaths happened in the first 5 years of life, and 47% of the under-five deaths occurred in the first month of life. Across all the SDG regions and in both high-income and low-income groups, over 80% of the deaths under 15 years of age happened in the first 5 years of life irrespective of the mortality level.
**Secondary outcomes**
*Skilled birth attendance*. In resource-limited settings, accessibility to a skilled attendant at the time of delivery is a vital lifesaving intervention for both mothers and babies. Not having access to this key assistance is a disadvantage to women's health because it could lead to the demise of the mother.
*Antenatal and post-natal attendance*. Having access to antenatal and post-natal care has a significant impact on infants' deaths and on trends in a maternal mortality through the provision of encouragement deliver with skilled birth attendant or in a health facility. When mothers miss post-natal clinic attendance after childbirth, it affects completion of the care and invariably contributes to maternal morbidity and mortality. Sub-Saharan Africa is consistently characterized by poor nature of post-natal clinic attendance.
*Vaccination and immunization coverage*. In spite of the recent success, almost 20% of the 8.8 million deaths under-five globally occurring each year are caused by vaccine-preventable disease. With the introduction of primary vaccination through the Expanded Program on Immunization (EPI) WHO, childhood vaccination was proved to be the most effective public health intervention. In spite of the evidence that vaccines are effective, many children in resource-limited areas such as Sub-Saharan Africa and Southern Asia either get vaccinated late or remain non-vaccinated.

### Methodological quality assessment

The overall quality related to the conduction of quantitative studies and reporting of the results is not always satisfactory (32). Studies of low methodological quality typically tend to report better treatment effects than studies of high quality (33–35). The Effective Public Health Practice Project (EPHPP) Quality Assessment Tool for Quantitative Studies was used to assess the quality of the eligible studies (36), since the EPHPP tool has proven to have a higher inter-rate agreement compared to the Cochrane Collaboration Risk of Bias tool when evaluating quantitative studies (37). The quality of each study was assessed in terms of selection bias, study design, confounders, blinding, data collection methods, and withdrawal and dropouts. A rating is assigned to each of the six components, and a final global rating (strong, moderate, weak) was then assigned. Studies with weak ratings were defined by the quality assessment tool as those with more than three weak component ratings. According to the recommendations by the Cochrane handbook (31) and the EPHPP Quality Assessment Tool for Quantitative Studies (36), studies of weak methodological quality were excluded from the final analysis to improve the quality of the results of this systematic literature review.

### Synthesis of the results

Data extracted on the characteristics and findings of the included studies were structured in form of a tabular summary. The findings were analyzed and organized according to study types, mHealth interventions and outcomes.

## Results

### Search results

**Figure 1:**
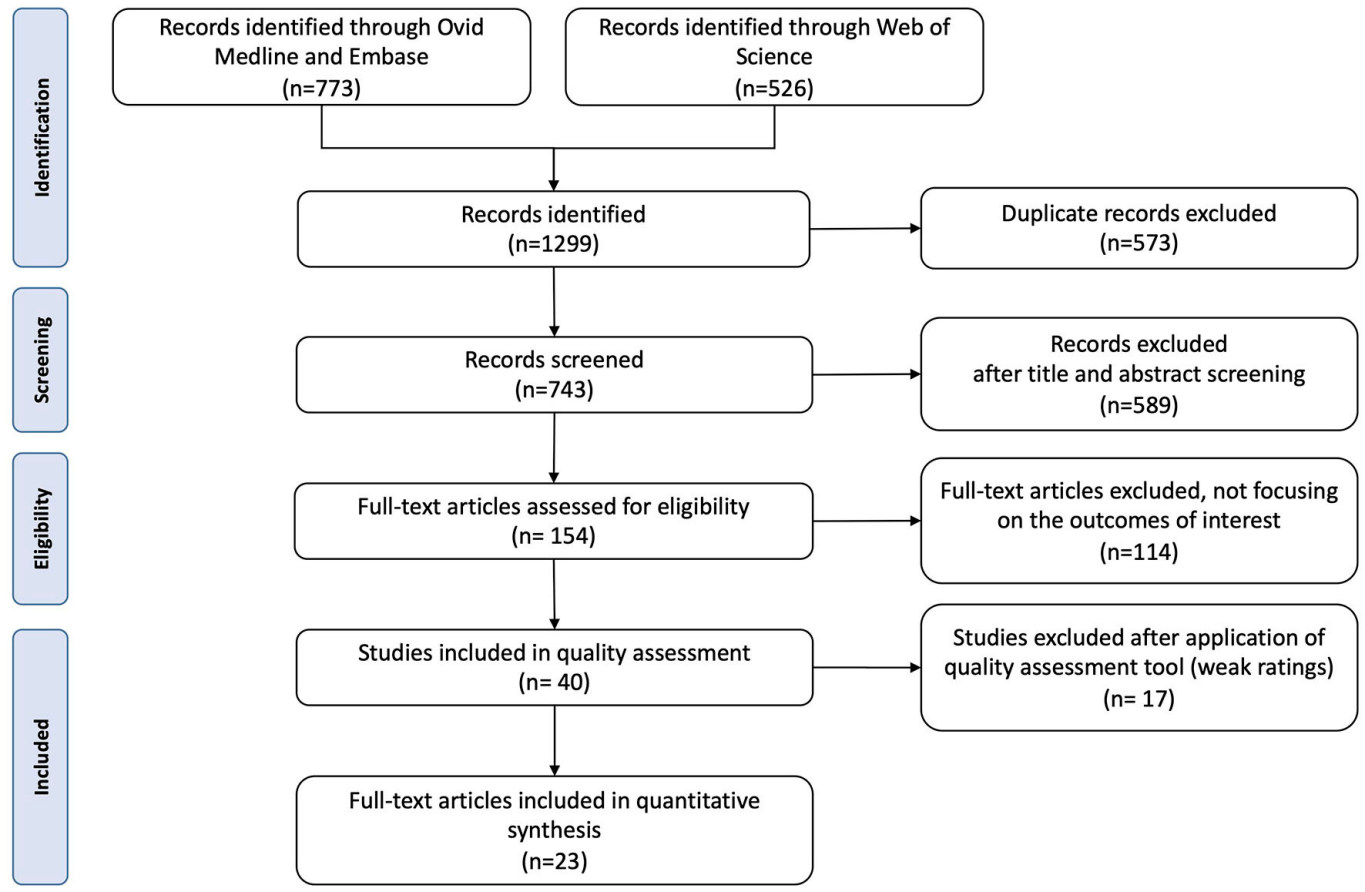
PRISMA flow diagram.

A total of 1,299 articles were identified from the search strategy (Figure 1). After removing duplicates and initial screening, 154 full-text articles were assessed for eligibility. Following further inspection and quality assessment (Supplementary Table 3), 23 articles were included in this review (19, 23, 38–58).

### Characteristics of the included studies

The characteristics of the included studies, including study type, population, interventions, level of care, and setting, were structured in a tabular summary (Table 3). There were 20 randomized controlled trials (RCTs), one non-randomized controlled trial, one quasi-experimental study and one retrospective cross-sectional survey. Sixteen studies were undertaken in Sub-Saharan Africa, while only seven were undertaken in Southern Asia. All studies except one were conducted in primary care settings.

**Table 3:** Characteristics of the included studies.

**First author**	**Year**	**Study type**	**Population**	**Intervention(s)**	**Level of care**	**Setting**
Alam (38)	2017	Retrospective cross-sectional survey	Mothers with last born child between 3 and 18 months	Mothers enrolled and exposed to the messages (SMS or voice) during pregnancy, delivery, information on nutrition for pregnant women and new mothers.	Primary care	Bangladesh
Atnafu (39)	2017	Randomized controlled trial	Health Extension Workers and Community Health Workers	Mobile phone equipped with short message service (SMS) based data-exchange software that stores maternal and child health forms and sends to central server. The server sends reminders of antenatal care visits, expected delivery date etc.	Primary care	Ethiopia
Bangure (40)	2015	Randomized controlled trial	Woman or caregiver recruited after delivery or 3rd and 7th day visits after delivery.	Intervention group: health education and SMS reminders. Control group: routine health education only.	Primary care	Zimbabwe
Brown (41)	2017	Randomized controlled trial	Mothers-infant pair. Infants aged 0–3 during the infants' first immunization visit	CALLS: cell phone calls reminding mothers to take child for immunizations.	Primary care	Nigeria
Dissieka (42)	2019	Randomized controlled trial	Motherchild pairs at the time of the child's BCG immunization visit (within 5 weeks of the child's birth)	Mothers (or caretakers) in the intervention group were provided SMS or voice message reminders, based on their preference, prior to each scheduled facility visit and two additional reminders in the event of non-attendance. Mothers in the control group were not provided any reminder messages.	Primary care	Ivory Coast
Ekhaguere (43)	2019	A randomized controlled trial	Parturient women and their healthy newborn infants delivered at state-run facilities	Windows software application (app) designed to send automated voice call text and email immunization reminders. Messaging and voice were sent by Twilio through the app.	Primary care	Nigeria
Gibson (44)	2017	Randomized controlled trial (4 groups)	Caregivers with a child aged <5 weeks yet to receive a first dose of pentavalent vaccine.	In the 3 intervention groups SMS reminders were sent with and without incentives (SMS reminders only, SMS + 75KES incentive, SMS + 200KES incentive) 3 days and the day before immunization at week 6, 10 and 14.	Primary care	Kenya
Hackett (45)	2018	Cluster randomized controlled trial	Community health workers	Smartphone application used by community health workers for client registration, home visit scheduling, time-tailored counseling prompts, automated referral and follow-up reminders. Paper-based protocols used for control group.	Primary care	Tanzania
Haji (46)	2016	Randomized controlled trial (3 groups)	Children aged <12 months presenting for their first dose of pentavalent vaccine	Two intervention groups with SMS reminders and stickers reminding guardians to return for second and third dose of vaccine.	Primary care	Kenya
Kazi (47)	2018	Randomized controlled trial	Child aged <2 weeks, parent or guardian	Intervention group: 4 SMS reminders + one-time standard verbal counseling. Control group: one-time standard verbal counseling.	Tertiary care	Pakistan
Lund (48)	2012	Cluster randomized controlled trial	Pregnant women who attended ANC.	Wired mothers: SMS to provide health education and appointment reminders and improve ANC, PNC and skilled delivery attendance.	Primary care	Zanzibar, Tanzania
Lund (49)	2014	Cluster randomized controlled trial	Pregnant women attending first ANC visit.	Wired mothers: SMS to provide health education and appointment reminders and improve ANC, PNC and skilled delivery attendance.	Primary care	Zanzibar, Tanzania
Lund (50)	2014	Cluster randomized controlled trial	Pregnant women attending first ANC visit.	Wired mothers: SMS to provide health education and appointment reminders and improve ANC, PNC and skilled delivery attendance.	Primary care	Zanzibar, Tanzania
Lund (51)	2016	Cluster randomized controlled trial	Health care workers in health care facilities.	Safe delivery App to increase health care workers' knowledge and skills in in neonatal resuscitation.	Primary care	Ethiopia
Modi (19)	2019	Cluster randomized controlled trial	Primary health center staff.	ImTeCH mobile-phone and web-based application to facilitate scheduling of home visits, screening for complications, counseling during home visits, and supportive supervision by primary health center staff.	Primary care	India
Murthy (52)	2020	Pseudo- randomized controlled trial	Pregnant women	mMITRA: age- and stage-based mobile phone voice messaging initiative for women, during pregnancy and up to 1 year after delivery.	Primary care	India
Murthy (53)	2019	Pseudo- randomized controlled trial	Pregnant women	mMITRA: age- and stage-based mobile phone voice messaging initiative for women, during pregnancy and up to 1 year after delivery.	Primary care	India
Nagar (54)	2018	Cluster randomized controlled trial (3 groups)	Mothers with an infant aged <6 months.	The intervention groups received NFC pendant and NFC pendant with voice call reminders. The control group received NFC sticker.	Primary care	India
Odeny (55)	2014	Randomized controlled trial	HIV-positive pregnant women aged >18 years.	SMS: eight text messages before delivery and six messages postpartum.	Primary Care	Kenya
Olajubu (23)	2020	Quasi-experimental study	Pregnant women at gestational age of 28–34 weeks.	A mobile health intervention software used to send educational and reminder messages (SMS) from the 35th week of pregnancy to 6 weeks after delivery.	Primary care	Nigeria
Oyeyemi (56)	2014	Case-control study	Pregnant women and health workers.	Free, Closed-Users' Group cell phones provided to pregnant women and health workers. Communication free of charge.	Primary care	Nigeria
Seth (57)	2018	Randomized controlled trial (3 groups)	Children aged ≤ 24 months and pregnant women.	Automated mobile phone reminders and compliance-linked incentives to the intervention groups. Routine verbal instructions about subsequent vaccination date to the control group.	Primary care	India
Shiferaw (58)	2016	Non-randomized controlled trial	Health workers and pregnant women.	Health workers in the intervention group used an application with SMS reminders for schedule visits during ANC, delivery and PNC.	Primary care	Ethiopia

ANC, antenatal care; NFC, Near Field Communication; PNC, post-natal care.

The vast majority of the studies reported mHealth interventions used for educational purposes. The most common form of delivery was SMS, used either alone as reminders or in combination with a mobile application (Table 4).

**Table 4:** Type of mHealth interventions for the included studies.

**Included studies (*n* = 23)**		
Mobile health intervention classification		
Education/awareness (behavior) (23 , 38 , 40 , 41 , 44 , 46 , 48 –50 , 52 , 53 , 55 , 58)	Communication and training (19, 23, 42, 43, 51–53, 58)	Registries/vital event tracking (36, 43, 45, 51–53, 58)
Outcomes of interest				
Neonatal mortality (50, 51)	Skilled birth attendance (38, 39, 42, 45, 48, 58)	Antenatal care attendance (39, 45, 49, 56, 58)	Post-natal care attendance (19, 23, 38, 55, 59)	Vaccination and immunization coverage (40–44, 46, 47, 49, 52, 54, 57)
Modes of intervention delivery				
SMS/voice message reminders (38–40, 42–44, 46–50, 55, 57)	Voice calls (41, 43, 54, 56)	Smartphone app (51)	App + SMS/voice reminders (19, 23, 43, 45, 52, 53, 58)	Data collection modules (19, 43, 57, 58)

### Synthesis of the study outcomes

#### Primary outcomes

##### Neonatal mortality

Only two studies reported neonatal mortality outcomes (Table 5). In a cluster RCT conducted in primary health care facilities in Zanzibar, the Wired Mothers intervention, consisting of a text message and free call voucher system linking women to the health system throughout their pregnancy, childbirth, and postpartum period, was associated with a significant reduction in perinatal mortality and a non-significant reduction in neonatal mortality (Odds Ratio (OR) 0.79) (50). In a RCT conducted on 73 primary healthcare facilities in Ethiopia, the use of a safe delivery app was associated with a non-significant decline in perinatal mortality (OR 0.76) (51).

**Table 5:** Synthesis of the outcomes on neonatal mortality.

**References**	**Intervention**	**Impact on neonatal mortality**
Lund et al. (50)	SMS reminders	Non-significant reduction in neonatal mortality in the intervention group (OR 0.79). Significant reduction in perinatal mortality in intervention group compared to control (19/1,000 vs. 36/1,000; OR 0.50).
Lund et al. (51)	App + SMS reminders	Non-significant lower perinatal mortality in the intervention group compared to the control group (14/1,000 vs. 23/1,000; OR 0.76)

OR, Odds Ratio.

##### Maternal mortality and under-five mortality

None of the studies included in this review reported results on maternal mortality and under-five mortality. No study from Southern Asia reported results on the primary outcomes.

#### Secondary outcomes

##### Skilled birth attendance

Six studies reported outcomes on skilled birth attendance (Table 6), which is a vital lifesaving intervention for both mothers and babies. Three studies delivering mHealth interventions based on SMS/voice message reminders only (38, 39, 48) demonstrated a positive impact on skilled birth attendance. In the Wired Mothers study, the intervention group receiving SMS reminders had a significantly higher percentage of women delivering with skilled birth personnel (60%) compared to the control group (47%) (48). The use of apps in combination with SMS or voice reminders has also been proven to significantly increase supervised institutional deliveries in three studies (45, 53, 58).

**Table 6:** Synthesis of the outcomes on skilled birth attendance.

**References**	**Intervention**	**Impact on skilled birth attendance**
Alam et al. (38)	SMS/voice message reminders	No significant association between exposure to phone messages during pregnancy and skilled birth attendance at home/health facility (RR 1.2)
Atnafu et al. (39)	SMS reminders	Delivery supervised by Health Extension Workers in the SMS group increased significantly compared to baseline. The control group experienced a significant reduction of deliveries supervised by Health Extension Workers.
Hackett et al. (45)	App + SMS reminders	Facility delivery in the mHealth intervention group was 74% compared to 63% in the control group at (OR 1.96)
Lund et al. (48)	SMS reminders	The intervention produced a significant increase in skilled delivery attendance amongst urban women (OR 5.73).
Murthy et al. (53)	App + voice reminders	The intervention group performed significantly better than controls on delivering in hospital (OR 2.5).
Shiferaw et al. (58)	App + SMS reminders + data collection module	Women in the intervention health centers significantly more likely to have institutional delivery compared to the control group (AOR 1.98).

AOR, Adjusted Odds Ratio; OR, Odds Ratio; RR, Relative Risk.

##### Antenatal and post-natal care attendance

Outcomes on ANC attendance (Table 7) and PNC attendance (Table 8) were reported in six studies (19, 39, 45, 49, 56, 58) and five studies (19, 23, 38, 55, 58), respectively. These studies showed that a variety of mHealth interventions delivered through simple mobile phone voice calls (56), SMS/voice reminders (38, 39, 49, 55) or apps/data collection modules (19, 23, 45, 58) can lead to improved access to ANC and PNC services. A study conducted in Ethiopia where SMS reminders for scheduled visits were sent to health workers demonstrated that women in the intervention health centers were more likely to attend at least four ANC visits (58). Attendance to four ANC visits was also associated with higher odds of PNC and delivery in a health facility where there is a greater chance of having access to a skilled birth attendant (58). Similarly, results from the SUSTAIN-MNCH study, where a smartphone-based application was designed to assist community health workers in Tanzania, showed that the uptake of ANC was a strong predictor of facility delivery (45).

**Table 7:** Synthesis of the outcomes on antenatal care attendance.

**References**	**Intervention**	**Impact on antenatal care attendance**
Atnafu et al. (39)	SMS reminders	The proportion of mothers receiving more than four ANC visits increased significantly in SMS group compared to baseline. The control group had a non-significant decline in ANC visits.
Hackett et al. (45)	App + SMS reminders	Perinatal home visit was 72% in the intervention group compared to 60% in the control group.
Lund et al. (49)	SMS reminders	Higher odds of receiving four or more ANC visits in the intervention group compared to the control group (44 vs. 31%; OR 2.39).
Modi et al. (19)	App + SMS reminders + data collection module	Significant improvement in coverage home visits in the intervention group during ANC period (adjusted effect size 15.7).
Oyeyemi et al. (56)	Voice calls	ANC utilization was significantly higher in the intervention area compared to the control area (43.4 vs. 36.7%)
Shiferaw et al. (58)	App + SMS reminders + data collection module	Women in the intervention centers significantly more likely to receive at least 4 ANC visits compared to controls (43.1 vs. 28.4%; AOR 1.98)

ANC, antenatal care; AOR, Adjusted Odds Ratio; OR, Odds Ratio; RR, Relative Risk.

**Table 8:** Synthesis of the outcomes on post-natal care attendance.

**References**	**Intervention**	**Impact on post-natal care attendance**
Alam et al. (38)	SMS/voice reminders	No significant association between exposure to phone messages during pregnancy and PNC visits (IRR 1.2)
Modi et al. (19)	App + SMS reminders + data collection module	The proportion of at least two visits of neonates in the first week of delivery was significantly higher in the intervention group compared to the control group (32.4 vs. 22.9%; adjusted effect size 10.2). Significant improvement in coverage home visits in the intervention group during PNC period (adjusted effect size 6.4).
Odeny et al. (55)	SMS reminders	Significant increase in women attending a post-partum visit in the intervention group compared to controls (19.6 vs. 11.8%; RR 1.66)
Olajubu et al. (23)	App + SMS reminders	The odds for utilizing four PNCs were about 11 times higher for women in the intervention group compared to controls (30.9 vs. 3.7%; AOR 10.869). For each of the recommended visits, the odds of utilization were significantly higher among the mothers in the intervention group.
Shiferaw et al. (58)	App + SMS reminders + data collection module	Women in the intervention centers significantly more likely to receive PNC visits compared to controls (41.2 vs. 21.1%; AOR 2.77).

AOR, Adjusted Odds Ratio; IRR, Incident Rate Ratio; PNC, post-natal care; RR, Relative Risk.

##### Vaccination/immunization coverage

Eleven studies had outcomes related to vaccination coverage (Table 9) (40–44, 46, 47, 49, 52, 54, 58). The vast majority of these studies delivered simple interventions consisting of SMS/voice message reminders (40, 42, 44–47, 54, 57) or voice calls (41). One study found that the intervention group receiving SMS reminders had a significantly lower vaccination dropout rate compared to the control group receiving routine reminders (OR 0.2) and a significantly higher vaccination coverage (46). In another study evaluating a cellphone-based reminder/recall strategy to improve childhood routine immunization, immunization compliance rate was 79.2% among the children in intervention group and 46.4% in the control group (41).

**Table 9:** Synthesis of the secondary outcomes on vaccination and immunization coverage.

**References**	**Intervention**	**Impact on vaccination and immunization coverage**
Bangure et al. (40)	SMS reminders	Immunization coverage in the intervention group significantly higher than in the control group at week 6 (93 vs. 82%), week 10 (96 vs. 80%), and week 14 (95 vs. 75%). Delay in immunization in the intervention group significantly less likely than to the control group.
Brown et al. (41)	Voice calls	Immunization compliance rate was 79.2% in the intervention group compared to 46.4% in the control group.
Dissieka et al. (42)	SMS/voice message reminders	Immunization coverage in the intervention group significantly higher than in the control group at pentavalent 1 (6 weeks) (86.6 vs. 76.1%, AOR 2.85), pentavalent 2 (10 weeks) (81.0 vs. 67.3%, AOR 2.80) and pentavalent 3 (14 weeks) (74.2 vs. 58.3%, AOR 2.68). Attendance at each visit was significantly higher in the intervention group compared to the control group.
Ekhaguere et al. (43)	App + SMS / voice reminders	The proportion of infants completing the 12-month immunization series in the intervention group was significantly higher compared to the control group (74 vs. 66%, RR 1.12). Timely receipts of immunization (within a week of expected date) were significantly higher in the intervention group compared to the control group (57 vs. 47%, RR 1.22).
Gibson et al. (44)	SMS reminders	The proportion of children achieving full immunization by 12 months of age was 82% (296/360) in the control group compared to 86% in the SMS intervention (332/388). Children in the SMS only group were significantly more likely to achieve full immunization (RR 1.04).
Haji et al. (46)	SMS reminders	Those who received text messages were less likely to drop out compared to controls (OR 0.2). Thirteen percent of the children vaccinated at 14 weeks in the SMS intervention group is attributed to SMS reminders.
Kazi et al. (47)	SMS reminders	PP analysis at 6 weeks demonstrated significantly higher immunization coverage in SMS intervention group compared to the control group (96.0 vs. 86.4%). Non-significant increase at week 10 and 14. ITT analysis at week 6, 10 and 14 indicated a non-significant increase in immunization coverage in the SMS intervention group compared to the control group.
Lund et al. (49)	SMS reminders	Non-significant improvement in vaccination uptake in the intervention group compared to the control group (72 vs. 56%, OR = 1.62)
Murthy et al. (52)	App + voice reminders	Women in the intervention group were 1.53 times more likely to report that their infant was fully immunized (OR 1.531). Babies born to women in the intervention group had 49% increased odds of receiving all their recommended immunizations as compared to controls (OR 1.485).
Nagar et al. (54)	Voice message reminders	Immunization completion within 2 months from registration time was higher in the two intervention groups (37.7 and 38.7%) compared to control (27.7%).
Seth et al. (57)	SMS reminders + data collection module	Median immunization coverage at enrollment was 33% in all groups and increased to 41.7, 40.1, and 50.0% in the control group, the group with mobile phone reminders, and the compliance-linked incentives group, respectively.

AOR, Adjusted Odds Ratio; ITT, intention-to-treat; OR, Odds Ratio; PP, per protocol; RR, Relative Risk.

## Discussion

### Summary of evidence

This study provides a comprehensive overview of mHealth interventions implemented in Sub-Saharan Africa and Southern Asia with outcomes addressing, directly or indirectly, the Target 3.1 and 3.2 of the SDG 3. We identified 23 relevant studies published from 2010 to 2020, which demonstrate a growing effort in evaluating the effects of mHealth interventions on maternal and child health outcomes. Low access to maternal health services, including skilled birth attendance at delivery, ANC attendance, PNC attendance, and vaccination and immunization coverage, represents a major cause of maternal and neonatal mortality. Interventions aimed at addressing those outcomes can therefore contribute to alleviate the burden of maternal and neonatal deaths targeted by the SDG 3. Despite few studies addressed mortality outcomes directly, the results from this systematic review showed that mHealth interventions are effective at changing the behavior of pregnant women, caretakers and health workers, and at increasing ANC attendance, PNC attendance, childhood vaccination/immunization rates, and skilled delivery attendance. Most of the mHealth interventions covered by this review were delivered with minimal resources and consisted of simple SMS or voice message reminders which are particularly suitable for resource-limited settings.

### mHealth impact on maternal, neonatal and under-five mortality

Only two studies (50, 51) reported results on neonatal mortality, while none of the included studies reported results on maternal and under-5 year mortality. The two studies with results on neonatal mortality found that SMS reminders for pregnant women and smartphone-based applications for education of health workers can be effective in reducing neonatal deaths in resource-limited countries. The evidence of the impact of mHealth interventions on neonatal mortality is, however, limited since it is based on two studies only, both conducted in the Eastern part of Sub-Saharan Africa. More studies conducted in the two SDG regions covered by this review, and especially in Southern Asia, are still needed to support the evidence of the effects on neonatal mortality. This is also necessary to avoid any divergence in the progress toward the SDGs between the two regions. Overall, only seven of the 23 studies included in this review were conducted in Southern Asia. A number of studies from Southern Asia were excluded from this review since they obtained a weak rating in the quality assessment (Supplementary Table 3).

### mHealth impact on skilled birth attendance

Skilled birth attendance is recognized as a crucial intervention to avert preventable obstetric complications, which are one of the main causes of maternal and perinatal mortality (59). The proportion of births or deliveries attended by skilled birth personnel is a vital indicator for harnessing the SDG Target 3.1. Access to skilled birth attendance is also addressed by the Global Strategy for Women's, Children's and Adolescents' Health 2016–2030 and by the framework for ending preventable maternal mortality 2015–2030 (60). The coverage of skilled birth attendants in Sub-Saharan Africa is lower compared to all other regions in the world (61). Moreover, the MDG tracking report (62) stated that the proportion of births in the presence of a skilled attendant in Sub-Saharan Africa increased only by 7% between 1990 (41%) and 2008 (48%).

Evidence from this systematic review demonstrated that mHealth interventions, such as the Wired Mothers, have the potential to support skilled birth attendance which, in turn, can contribute to reduce the global maternal mortality (48). Other studies concluded that mHealth interventions can support behavioral change and influence women's choice of delivery attendance (63, 64). The Safe Delivery Application study conducted in Ethiopia demonstrated that mHealth interventions can improve knowledge and skill acquisition of healthcare workers as well as quality care during emergencies in child birth (51). Such findings are instrumental in addressing the challenges in the provision of quality care due to the absence of continuing education programs. Findings from another study conducted in Ethiopia on the use of a mHealth intervention to improve delivery and PNC (58) supported an earlier study conducted in Zanzibar testing the ability of SMS reminders to improve skilled birth delivery at births (48).

### mHealth impact on antenatal and post-natal care attendance

Having access to ANC and PNC has a significant impact on neonatal mortality as well as an indirect role in reducing maternal mortality by encouraging women to deliver with assistance of a skilled birth attendant or in a health facility (65). Interventions encouraging frequent ANC contacts allow pregnant women to prepare for delivery and be acquainted with educational information on the warning signs of poor maternal or infant health during pregnancy and childbirth. Likewise, interventions aimed at promoting PNC allow mothers and babies to be assessed during visits and can mitigate the risks of maternal and infant morbidity and mortality, which are highest in the days and weeks following childbirth (66–68). mHealth interventions have the potential to support the uptake of these maternal and child health services, thus contributing to reduce regional differences in infant and maternal mortality.

The Wired Mothers (48) and the SUSTAIN-MNCH (45) studies indicated that mHealth interventions delivered through smartphones can contribute to attain the World Health Organization's (WHO) recommendation of four ANC visits in the course of pregnancy (at weeks 14, 24, 30, and 36) and delivery at a health facility (69, 70). Such interventions are especially useful in disadvantaged settings burdened with poverty and low rates of facility delivery. Additionally, the SUSTAIN-MNCH intervention and the Safe Delivery Application confirmed the importance of training of health workers through smartphone applications to enhance uptake of health facility delivery and skills improvement on newborn resuscitation, thus supporting the global effort in reducing neonatal mortality. The results from the Safe Delivery Application support the findings from a review showing that neonatal resuscitation reduces neonatal and perinatal mortality (71). Moreover, ensuring that every child delivery is done in the presence of skilled health personnel is a key to reduce the global maternal deaths (72). The current systematic review showed that access to skilled birth delivery and health facility delivery, as part of mHealth interventional strategies in the continuum of care, can contribute to reduce mortality rates as addressed by the SDG Target 3.1 and 3.2.

### mHealth impact on vaccination and immunization coverage

A UNICEF report in 2015 acknowledges childhood diseases as the cause of most neonatal and under-five deaths in Sub-Saharan Africa (73). A simple and effective way of protecting children and minimizing the spread of diseases is immunization. The main target of the immunization agenda 2030 is to make immunization achievable for everyone, everywhere, at every age, by 2030. A number of studies have confirmed that immunization is a cost-effective public health intervention capable of contributing significantly to the SDG 3.2 (i.e., end avoidable deaths of newborns and children under five) (74–76).

The results from this review are indicative of how simple mHealth interventions, including SMS reminders (40, 44, 46), voice message reminders (42, 52, 54) and voice calls (41), are effective at improving vaccination and immunization coverage in resource-limited settings where coverage is low, such as Sub-Saharan Africa and Southern Asia. The available evidence also shows that mHealth interventions can contribute to reduce delays in immunization and improve immunization compliance rate (40, 41), thus increasing adherence to timely receipts of immunization and immunization completeness (43, 54). This will ultimately contribute to the Global Vaccine Action Plan (GVAP), which aims to achieve at least 90% national vaccination coverage and herd immunity level of immunization coverage where the chain of disease transmission is broken. Improved vaccination and immunization coverage will, in turn, support the SDG Target 3.2 of ending avoiding deaths of newborns and children under-5 years of age.

### Limitations

According to the scope of this systematic literature review and its eligibility criteria, only papers published in peer-reviewed journals were included. As a consequence, other sources which might include useful results on maternal and child mortality, such as reports from non-profit organizations or white or gray literature, were excluded. Moreover, the study included only papers published in English. It is possible that studies reporting results of mHealth interventions conducted in Sub-Saharan Africa and Southern Asia were published in another language than English. It is also worth noting that there was an overlap among some of the studies included in this review. In particular, three papers reporting on use of SMS reminders were linked to the same program in Zanzibar, Tanzania (48–50). The generalizability of the findings from this review is affected by the variety of the mHealth interventions described in the included studies.

### Policy implications

This systematic literature review provides a useful contribution toward the role of mHealth interventions in achieving the UN's SDG 3. The results showed an increasing number of evidence-based interventions aimed to make pregnancy and childbirth safer for both mother and child by alleviating the burden of maternal and neonatal deaths. The findings from this study can inform policy makers and serve as a basis to provide recommendations on the implementation of mHealth interventions in resource-limited settings, including SMS messages to improve maternal health-seeking behavior and reminders to caretakers in the health delivery structure.

With most maternal, neonatal and child deaths taking place in Sub-Saharan Africa and Southern Asia, a need for action is required to achieve the SDG Target 3.1 and 3.2. If innovative interventions supporting maternal, neonatal and child health are implemented in the healthcare delivery system, more than half of the global 56 million deaths under-5 year could potentially be prevented between 2018 and 2030 in Sub-Saharan Africa and Southern Asia (77). The potential presented by mHealth interventions is promising and offers new hope for the future. The progress demonstrated in the two SDG regions included in this systematic review shows that the maternal and child health can be improved through mHealth interventions aimed to increase access to skilled delivery attendance, vaccination coverage, immunization uptake, ANC and PNC. Increasing women's access to quality care, from the antenatal stage to the post-natal period, also contribute to improving equity and reducing health disparities between high-income and low-income countries. This ultimately contributes to a growing evidence that mHealth can support the SDG 3 (78) by reducing maternal and neonatal mortality in the post 2015 agenda of the UN.

## Conclusions

The results from this systematic review are indicative of how simple mHealth educational interventions based on SMS and voice message reminders, used either alone or in combination with a mobile application, can support behavior change of pregnant women and training of health workers and are effective at improving skilled birth attendance, ANC and PNC attendance, and vaccination coverage. At the primary health care level, mHealth interventions can play a pivotal role in increasing access to quality care in a decentralized health care system. This is especially important in resource-limited settings, such as Sub-Saharan Africa and Southern Asia, where an acceleration of progress is needed to address the burden of maternal mortality and neonatal mortality. Higher quality studies addressing the role of mHealth in reducing maternal and child mortality in resource-limited settings are needed, especially in Southern Asia.

## Data availability statement

The original contributions presented in the study are included in the article/Supplementary material, further inquiries can be directed to the corresponding author/s.

## Author contributions

Conceptualization, methodology, validation, and writing—review and editing: EB, MJ, and PZ. Data curation, visualization, and writing—original draft: EB. Investigation: EB and PZ. Supervision: MJ and PZ. All authors contributed to the article and approved the submitted version.

## Funding

This study was initiated and managed by UiT The Arctic University of Norway as part of a master's degree program in Telemedicine and E-health. Access to commercial databases was granted through the UiT library.

## Conflict of interest

The authors declare that the research was conducted in the absence of any commercial or financial relationships that could be construed as a potential conflict of interest.

## Publisher's note

All claims expressed in this article are solely those of the authors and do not necessarily represent those of their affiliated organizations, or those of the publisher, the editors and the reviewers. Any product that may be evaluated in this article, or claim that may be made by its manufacturer, is not guaranteed or endorsed by the publisher.
