# Supplementary material for: mHealth interventions to reduce maternal and child mortality in Sub-Saharan Africa and Southern Asia: A systematic literature review
Source: Front Glob Womens Health. 2022 Aug 25;3:942146. doi: 10.3389/fgwh.2022.942146 (PMC9453039; doi:10.3389/fgwh.2022.942146)
Supplement: Supplementary file 1 [file Table_1.DOCX]

Supplementary Material

**Supplementary table 1.** Search terms under the PICOS framework.

| - Population | - Interventions | - Outcomes | - Setting |
| --- | --- | --- | --- |
| - Pregnant women, matern*, - gestation, - women, - mother, - Infant, - Infant health - infant, - Newborn, - newborn, - neonat*, - Perinat*, Child, - Preschool - Health facilit* | - mHealth, - mobile health, - m-Health, - mobile phone, - Cell phone, Smartphone, - mobile application, - short message service, - Text messaging, - mobile device | - Perinatal death, Infant death, - Perinatal mortality, - Infant mortality, - Pregnancy complication, - Child death, Neonatal mortalit*, - Neonatal death, Mortalit* death* - Under five mortality, - Antenatal attend* - Postnatal care, Postnatal visit* - Vaccinat*, Immunizat* - Civil registration, Vital statistics - Skilled birth attend* | - Africa south of the Sahara, - Sub-Saharan Africa - Afghanistan, - Bangladesh, - Bhutan, - India, - Iran, - Maldives, - Nepal, - Pakistan, - Sri Lanka |
