# Supplementary material for: mHealth interventions to reduce maternal and child mortality in Sub-Saharan Africa and Southern Asia: A systematic literature review
Source: Front Glob Womens Health. 2022 Aug 25;3:942146. doi: 10.3389/fgwh.2022.942146 (PMC9453039; doi:10.3389/fgwh.2022.942146)
Supplement: Supplementary file 2 [file Table_2.DOCX]

Supplementary Material

**Supplementary table 2.** Data extraction form.

| - First author - Year of publication - Study title - Objectives - Study type - Population / participants - Intervention(s) and control - Level of care (e.g. primary, secondary or tertiary level) - Country / SDG region - Primary outcomes (maternal mortality, neonatal mortality, under-five mortality) - Secondary outcomes (skilled birth attendance, antenatal care attendance, postnatal care attendance, vaccination/immunization coverage) - Quality assessment rating |
| --- |
