# Supplementary material for: mHealth interventions to reduce maternal and child mortality in Sub-Saharan Africa and Southern Asia: A systematic literature review
Source: Front Glob Womens Health. 2022 Aug 25;3:942146. doi: 10.3389/fgwh.2022.942146 (PMC9453039; doi:10.3389/fgwh.2022.942146)
Supplement: Supplementary file 3 [file Table_3.DOCX]

Supplementary Material

**Supplementary Table 3**. Quality assessment of potentially eligible studies by the EPHPP Quality Assessment Tool for Quantitative Studies (1= Strong, 2 = Moderate, 3 = Weak, N/A = Not Applicable).

| **Reference, author (year)** | **Selection Bias** | **Study design** | **Confounders** | **Blinding** | **Data collection methods** | **Withdrawal and dropouts** | **FINAL RATING** |
| --- | --- | --- | --- | --- | --- | --- | --- |
| Adanikin (2014) | 2 | 2 | 3 | 3 | 1 | 3 | Weak |
| Afzal (2017) | 3 | 2 | 3 | 2 | 1 | 2 | Weak |
| Alam (2017) | 2 | 2 | 1 | 2 | 1 | 2 | Strong |
| Amoah (2016) | 3 | 3 | 3 | 3 | 2 | N/A | Weak |
| Amoakoh (2019) | 2 | 1 | 3 | 3 | 2 | 1 | Weak |
| Atnafu (2017) | 1 | 1 | 1 | 2 | 1 | 1 | Strong |
| Bangure(2015) | 2 | 1 | 1 | 2 | 2 | 1 | Strong |
| Benski (2020) | 2 | 3 | 3 | 3 | 1 | 3 | Weak |
| Brown (2017) | 1 | 1 | 3 | 1 | 1 | 1 | Moderate |
| Carmicheal (2019) | 1 | 1 | 3 | 3 | 2 | 2 | Weak |
| Coleman (2017) | 3 | 2 | 1 | 3 | 1 | 3 | Weak |
| Coleman (2020) | 2 | 2 | 1 | 3 | 3 | 3 | Weak |
| Dissieka (2019) | 1 | 1 | 1 | 1 | 1 | 1 | Strong |
| Ekhaguere (2019) | 1 | 1 | 1 | 1 | 1 | 1 | Strong |
| Gibson (2017) | 1 | 1 | 1 | 3 | 2 | 2 | Moderate |
| Hackett (2018) | 1 | 1 | 2 | 3 | 1 | 2 | Moderate |
| Haji (2016) | 2 | 1 | 1 | 3 | 2 | 2 | Moderate |
| Hategeka (2019) | 2 | 2 | 3 | 3 | 1 | 3 | Weak |
| Ibraheem (2017) | 2 | 3 | 1 | 3 | 3 | N/A | Weak |
| Jennings (2015) | 3 | 3 | 3 | 2 | 3 | N/A | Weak |
| Kazi (2018) | 2 | 1 | 1 | 3 | 1 | 1 | Moderate |
| Lund (2016) | 2 | 1 | 1 | 2 | 1 | 1 | Strong |
| Lund (2012) | 2 | 1 | 1 | 1 | 1 | 1 | Strong |
| Lund (2014) | 2 | 1 | 1 | 3 | 1 | 1 | Moderate |
| Lund (2014) | 2 | 1 | 1 | 3 | 1 | 1 | Moderate |
| Modi (2016) | 2 | 3 | 3 | 3 | 2 | N/A | Weak |
| Modi (2019) | 1 | 1 | 1 | 2 | 1 | 1 | Strong |
| Murthy (2019) | 2 | 2 | 3 | 2 | 1 | 2 | Moderate |
| Murthy (2020) | 2 | 2 | 3 | 2 | 1 | 2 | Moderate |
| Mushamiri (2015) | 1 | 2 | 3 | 3 | 1 | N/A | Weak |
| Nagar (2018) | 2 | 1 | 1 | 2 | 3 | 2 | Moderate |
| Odeny (2014) | 2 | 1 | 1 | 3 | 1 | 1 | Moderate |
| Oladepo (2020) | 1 | 1 | 1 | 3 | 3 | 3 | Weak |
| Olajubu (2020) | 1 | 2 | 1 | 3 | 1 | 1 | Moderate |
| Oyeyemi (2014) | 2 | 2 | 3 | 2 | 1 | N/A | Moderate |
| Prinja (2017) | 2 | 3 | 1 | 3 | 3 | N/A | Weak |
| Seth (2018) | 2 | 1 | 1 | 3 | 1 | 1 | Moderate |
| Shiferaw (2016) | 3 | 1 | 2 | 3 | 1 | 1 | Moderate |
| Tang (2019) | 1 | 3 | 1 | 3 | 1 | 3 | Weak |
| Uddin (2016) | 2 | 3 | 1 | 1 | 3 | N/A | Weak |
